# Over view for the truth of COVID -19 pandemic: A guide for the Pathologists, Health care workers and community’

**DOI:** 10.12669/pjms.36.COVID19-S4.2519

**Published:** 2020-05

**Authors:** Mulazim Hussain Bukhari, Khalid Mahmood, Syeda Ailia Zahra

**Affiliations:** 1 Mulazim Hussain Bukhari, Head of the Department of Pathology, Department of Pathology, University College of Medicine, University of Lahore, Lahore, Pakistan; 2 Khalid Mahmood Pathology, Department of Microbiology, Department of Pathology, University College of Medicine, University of Lahore, Lahore, Pakistan; 3 Syed Ailia Zahra, Student A level 26 A Divine Garden Airport Road, Lahore

**Keywords:** COVID-19, Laboratory Staffs, Pathologists, Pandemic, PPE, SARS-CoV2

## Abstract

Pakistan is in the grip of COVID-19, due to severe acute respiratory syndrome coronavirus-2 (SARS-CoV-2) since 26 February 2020, and the number of infected people and mortality is rising gradually. The health workers, doctors, pathologists and laboratory staff are front line fighters who are facing the risk. Few things are important for public and health workers, human behavior is at the core of preparedness and response i.e, personal protective measures, (handwashing, face masks, respiratory etiquette, surface and objects cleansing), social distancing and travel measures because the virus spreads through the respiratory channels, eyes, nose and mouth. While working in the Pathology labs, use the personal protection equipment (PPE), during the work in the duty. Avoiding the over duties and long shifts. It is good to keep the immune system healthy by taking a healthy balanced diet, vitamin supplements, and a night of proper sleep. It is also important to avoid taking food during duties and avoid making close contact without wearing safety dress.

Pakistan is a developing country with a population of more than 220 million, with low hygienic facilities and insufficient awareness about the current Outbreak of COVID-19. Due to the global available date, the virus has been widespread since December 2019. “How can we go on to make an standard operating procedure (SOP) list off “what we have to do and what are the precautions that we should adopt to save ourselves, staff working in the labs, is very important.” It is important to cut off human-to-human transmission including reducing the spread of infection among close contact and workers of the health care, ceasing the transmission boosting routes, and preventing the pandemic. The social behaviour is very important to controll the outbreak of this virus. (Figure 1)

**Fig.1:**
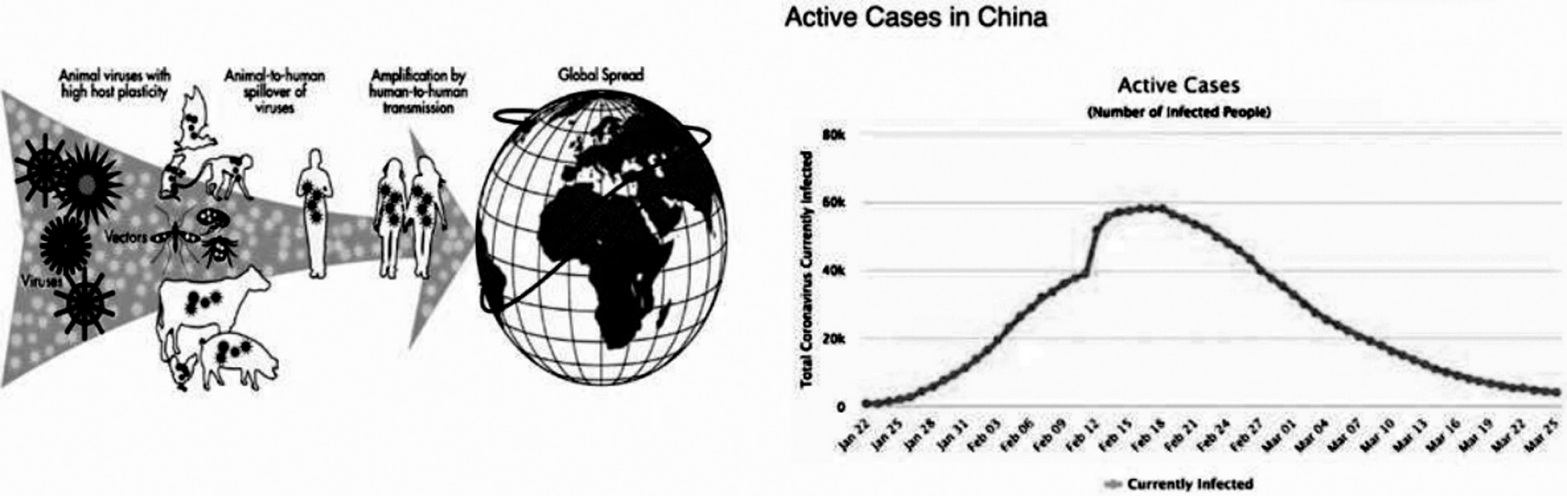
Human behaviour is at the core of Pandemic preparedness and response, How china have controlled the outbreak.

At laboratory level, classification is done in terms of arrangements to keep any virus safe. Conjecture is that the outbreak of coronavirus was spread from Wuhan, that the SARS-CoV2 may have leaked from her institute, where a lab certified as BSL-4, the highest level for handling dangerous pathogens, opened three years ago. How it is possible that an outbreak has taken place, is this a biological terrorism? Is this a part of the biological war? All these question raise and the world should unite to search the answers. How can we save our Pathology staff, because we are the people who are directly dealing with the infected material, the outbreak has provided some common sense tips that you have likely seen elsewhere by now, it also highlighted what should be done to keep the lab safe.^1^-^4^

The initial important points included in SOP are washing of hand for 20 seconds, after touching anything in general which has been touched or used by others in a day many times, such as door handles, stair bars, light switches, elevator buttons, ATM Machines etc. In labs pippets, microscopes, knobs and all other instruments and machinery which are used should not be touched before being cleaned properly and everything should be used wearing gloves. The PCR areas are very sensitive hence should not be used without adopting the SOP for these labs. Sample collection of the suspected people should only be collected wearing PPE protected gloves, goggles and N95 masks. These samples should be transported carefully to the PCR labs. By following the advice of National Institute of Health (NIH) and Centers for Disease Control (CDC). Everything that is associated with infected people will be contaminated and potentially infectious. The virus is on the surfaces and one will not be infected unless one has unprotected face is directly coughed or sneezed upon.^2^,^6^

When you are in labs use disposable overalls, disposable gloves or paper towels especially when you use these things or lift the gasoline dispenser. The restrooms, grocery places, baby carts at commercial places, offices are commonly used by hundreds of people daily, at such places, when you need to open the doors, use your fist to turn the opening position and use your hip or elbow to open it to avoid grasping the handle with your hand. If there is no other way to open the door, then wash hands before starting the work. Use the latex or nitrile latex disposable gloves for use.

Use alcohol-based hand sanitizer whenever you return home from your duty activity that involves locations where other people have been present. Installation of sanitizers at your home entrances, and even in your cars for use after getting gas or touching other contaminated objects when you can’t immediately wash your hands is advised. One should change clothes and take a hot bath after coming home from work. Keep the things outside which you have used at work.^5^

When you cough or sneeze, always use disposable tissue papers and then dump them or discard properly. Avoid touching the clothing near your wrists, use the clothes up to the elbow because the clothing on your writs will contain an infectious virus that can be passed up in the next few days. Avoid shaking hands, touching your face, hugging the guests, relatives kissing the babies as a cultural routine in different countries, in Arabs and in Saraiky culture in Central Pakistan, South Punjab.^7^

This current outbreak of COVID-19 has repeated the memories of the previous outbreaks of SARS-CoV (Guangdong, China in 2002-2004), MERS CoV (Jeddah, Saudi Arabia in 2012-2013) resulting in mortalities 858 (37%) and 744 (10%) respectively. It is not yet clear whether the disease was first transmitted to humans by animals or through contamination levels by the contact.^8^

However, it is now clear that the COVID-19 is spreading through droplets by coughing and sneezing, it cannot reach if the person is at a distance of two meters from infected person but if the air is not infecting you, but contaminating, all the surfaces where these droplets are striking can infect others trough touching the infected surface for about a week on average.^4^-^10^

The current virus can infect the lower respiratory region; due to having cell receptors for lungs. The route of infection is through nose, mouth, and eyes, through coughing, sneezing, touching the infected surfaces and helping the virus to enter in the mucous membrane, through hands. These infections may be serious in patients whose immune system is compromised i.e., diabetes, blood pressure, Tuberculosis (TB), chronic obstructive diseses (COPDs), hepatitis or any heart disease, they need to be more cautious. Younger children and the elderly need more care. Avoid going to the fish or meat markets and stay away from pets and animals kept at home. Be careful about the family and relatives of people who have had the virus.^9^

The use of disposable surgical masks and spectacles prevent the virus in a direct sneeze from getting into your nose or mouth as well as it can keep you safe from touching the eyes, nose or mouth, because unintentionally we touch these areas many time a day and eat things without washing the hands. There will be no drugs or vaccines available this year to protect us or limit the infection within us. Only symptomatic support is available.” But there are certain important things to boost your immunity, like vitamic C and D. The use of Remdesivir (antiebola), Favipiravir (antiflu), lopinavir (anit HIV) hyrdoxy choloquine, chroloquine phasphate, (antimalarial) azithromycin, (antibiotic), interferon beta, antibody therapy all are still not approved by the FDA, but the use of these drugs has shown better recovery in the patients suffering from life threatening COVID19.^10^

It has been suggested that washing your mouth, throat, and the larynx with light warm salt mix water by doing gargles, every day in the morning after breakfast before leaving to your laboratories, every day may not allow the virus to attach your mucous membrane. Nanshan Z* (SARS coronavirus in 2003) suggested simple ways to prevent pneumonia by rinsing throat with light salt mix hot water before going to the hospital or other public places (and do the same after you return home), may stop the invasion of this new virus. The method is as follows: take a mouthful of dilute salty light hot water, raise the head; let the salt mixed water stay around the throat area, open the mouth slightly and exhale it slowly, let the air bubbling through the water in the throat by gargling with “*haa”* sound, then spit out the water after a few seconds and repeat the process 3-5 times. Because viruses or bacteria enter in the nasopharynx through the nostrils, by attaching the mucosa, the diluted salt mixed light hot water can kill them on the spot at about the temperature of 26-27ºC, thereby achieving the purpose of preventing infection. During last SARS, the method was taught to the students to prevent the attack of this virus, and none of the students in the class got cold, cough or fever. This method is simple, effective, easy to do. It was just his observation which may require perseverance.^11^

During your duties in laboratories, imbibe the Zinc or vitamin C containing tablets which may help in stopping any virus and especially these days Coronavirus (and most other viruses) from multiplying in the throats and nasopharynx as they improve the immunity. The Zinc is required for the normal functioning of living cells. And vitamin C contributes to immune defense by supporting various cellular functions of the immune system. The Zinc acts as co-factor, for the enzymes involved in the cell cycle for the DNA replication and transcription, i.e., plays a significant role in the growth and tissue maintenance.^12^

A Pathologist, Eby GA, has conducted a clinical trial on the use of zinc gluconate lozenges for the treatment of the flu, according to his observations, the zinc gluconate improves the human immune function by protecting and curing the common cold, as a nonspecific antiviral effect. In so many studies, zinc is supplemented to patients under immunosuppressive therapy especially for cancer. Once George Eby used a zinc gluconate tablet for his 3-year-old daughter, who was going under chemotherapy for acute T-cell lymphocytic leukemia. He used this to recover her from a cold within several hours after dissolving in her mouth rather than swallowing this tablet.^13^

Now if you use these zinc lozenges several times each day when you begin to feel any “cold-like” symptoms. It is best to lie down and let the lozenge dissolve in the back of your throat and nasopharynx. Cold-Eeze lozenges could work as a ‘silver bullet’ that would kill coronavirus. The zinc will inhibit the replication of many viruses, including coronaviruses. We cannot claim but can expect that the COVID-19 may be inhibited similarly. Take vitamin A, D, and Vitamin C, because the literature shows these vitamins may support the human immune system.^14^

The disease has become a pandemic, with lots of moralities, but there is no need to panic, because 80% of the cases, it is mild, 14-15% moderate and only 5% severe, with the mortality rate 1.5-5% in different countries. Following the precautionary measures advised by WHO and improving immune system with good diets, it can be controlled. In COVID19 so far, the death toll has not shown the trend than the other pandemics of history like Spanish flu, plague, Zika virus, and Ebola virus. What should be done to keep the immunity strong and take special care of cleanliness?^12^,^14^-^16^

## CONCLUSION

Hand washing, using face masks, adopting respiratory etiquette, and cleaning surface and objects, social distancing and travel measures can protect us from the COVID19. The identification and follow up of the contacts, to reduce the community spread. Creation of aware of the infection prevention, implementation of health measures and risk communication to the public is important in current conditions rather making lock down. The health workers, laboratory staff, pathologists should use surgical mask, and PPE kits, N95 masks during dealing with the patients or material of the infected person. Take good healthy food, optive drugs, and vitamin supplements to maintain proper health.

Few things are important for public and health workers, like handwashing, face masks, respiratory etiquette, surface and objects cleansing), social distancing and travel measures because the virus spreads through the respiratory channels, eyes, nose and mouth. While working in the Pathology labs, use the personal protection equipment (PPE), during the work in the duty. Avoiding the over duties and long shifts. It is good to keep the immune system healthy by taking a healthy balanced diet, vitamin supplements, and a night of proper sleep. It is also important to avoid taking food during duties and avoid making close contact without wearing safety dress.
